# Accumulation of antimicrobial resistance genes in wild chimpanzees

**DOI:** 10.1093/ismejo/wrag179

**Published:** 2026-07-09

**Authors:** Coch Tanguy Floyde Tanga, Markus Ulrich, J Benjamin Stielow, Elias Eger, Katharina Schaufler, Stefan E Heiden, Michael Schwabe, Liran Samuni, Cathy Crockford, Tobias Deschner, Patrice Makouloutou-Nzassi, Rodrigue Mintsa-Nguema, Roman M Wittig, Sébastien Calvignac-Spencer, Fabian H Leendertz, Jan F Gogarten, Ariane Düx

**Affiliations:** Department of Biology and Animal Ecology, Research Institute for Tropical Ecology (IRET/CENAREST), Libreville BP 13354, Gabon; Faculty of Mathematics and Natural Sciences, University of Greifswald, 17489 Greifswald, Mecklenburg-Vorpommern, Germany; Helmholtz Institute for One Health (HIOH), Helmholtz Centre for Infection Research (HZI), 17489 Greifswald, Mecklenburg-Vorpommern, Germany; Helmholtz Institute for One Health (HIOH), Helmholtz Centre for Infection Research (HZI), 17489 Greifswald, Mecklenburg-Vorpommern, Germany; Helmholtz Institute for One Health (HIOH), Helmholtz Centre for Infection Research (HZI), 17489 Greifswald, Mecklenburg-Vorpommern, Germany; Department of Hygiene and Medical Microbiology, Medical University of Innsbruck, 6020-A Innsbruck, Austria; Helmholtz Institute for One Health (HIOH), Helmholtz Centre for Infection Research (HZI), 17489 Greifswald, Mecklenburg-Vorpommern, Germany; Helmholtz Institute for One Health (HIOH), Helmholtz Centre for Infection Research (HZI), 17489 Greifswald, Mecklenburg-Vorpommern, Germany; University Medicine Greifswald, 17489 Greifswald, Mecklenburg-Vorpommern, Germany; Helmholtz Institute for One Health (HIOH), Helmholtz Centre for Infection Research (HZI), 17489 Greifswald, Mecklenburg-Vorpommern, Germany; Helmholtz Institute for One Health (HIOH), Helmholtz Centre for Infection Research (HZI), 17489 Greifswald, Mecklenburg-Vorpommern, Germany; Cooperative Evolution Laboratory, German Primate Center, Leibniz Institute for Primate Research, 37083 Göttingen, Niedersachsen, Germany; Taï Chimpanzee Project, Centre Suisse de Recherches Scientifiques, 01 BP 1303 Abidjan, Côte d’Ivoire; Taï Chimpanzee Project, Centre Suisse de Recherches Scientifiques, 01 BP 1303 Abidjan, Côte d’Ivoire; The Ape Social Mind Laboratory, Institut des Sciences Cognitives, CNRS University of Lyon, 69500 Lyon, Bron, France; Comparative BioCognition, Institute of Cognitive Science, University of Osnabrück, 49076 Osnabrück, Niedersachsen, Germany; Max Planck Institute for Evolutionary Anthropology, 04103 Leipzig, Sachsen, Germany; Department of Biology and Animal Ecology, Research Institute for Tropical Ecology (IRET/CENAREST), Libreville BP 13354, Gabon; Department of Biology and Animal Ecology, Research Institute for Tropical Ecology (IRET/CENAREST), Libreville BP 13354, Gabon; Taï Chimpanzee Project, Centre Suisse de Recherches Scientifiques, 01 BP 1303 Abidjan, Côte d’Ivoire; The Ape Social Mind Laboratory, Institut des Sciences Cognitives, CNRS University of Lyon, 69500 Lyon, Bron, France; Faculty of Mathematics and Natural Sciences, University of Greifswald, 17489 Greifswald, Mecklenburg-Vorpommern, Germany; Helmholtz Institute for One Health (HIOH), Helmholtz Centre for Infection Research (HZI), 17489 Greifswald, Mecklenburg-Vorpommern, Germany; Faculty of Mathematics and Natural Sciences, University of Greifswald, 17489 Greifswald, Mecklenburg-Vorpommern, Germany; Helmholtz Institute for One Health (HIOH), Helmholtz Centre for Infection Research (HZI), 17489 Greifswald, Mecklenburg-Vorpommern, Germany; Taï Chimpanzee Project, Centre Suisse de Recherches Scientifiques, 01 BP 1303 Abidjan, Côte d’Ivoire; Helmholtz Institute for One Health (HIOH), Helmholtz Centre for Infection Research (HZI), 17489 Greifswald, Mecklenburg-Vorpommern, Germany; Department of Applied Zoology and Nature Conservation, Zoological Institute and Museum, University of Greifswald, 17489 Greifswald, Mecklenburg-Vorpommern, Germany; Helmholtz Institute for One Health (HIOH), Helmholtz Centre for Infection Research (HZI), 17489 Greifswald, Mecklenburg-Vorpommern, Germany; Taï Chimpanzee Project, Centre Suisse de Recherches Scientifiques, 01 BP 1303 Abidjan, Côte d’Ivoire

**Keywords:** Antibiotic resistance genes, longitudinal analysis, wildlife, West Africa

## Abstract

Emergence of antimicrobial resistance (AMR) is a critical public health issue. The unregulated use of antibiotics in some regions of Sub-Saharan Africa make AMR emergence a prominent problem. Complex human-animal interfaces in these regions are hypothesized to create opportunities to transmit AMR, both in the form of resistant bacteria and mobile genetic elements carrying antibiotic resistance genes (ARGs). However, assessing the spread of ARGs into wildlife populations is complicated by naturally occurring resistance. Here, we use a longitudinal approach to explore whether the widespread use of antimicrobial compounds in West Africa was accompanied by an increase of ARGs in wild chimpanzees (*Pan troglodytes verus*) in Taï National Park (TNP), Côte d’Ivoire, the largest remaining piece of primary rainforest in West Africa. We analyzed 410 fecal samples from three groups collected over 17 years using hybridization capture and high-throughput sequencing to screen for over 2000 ARGs. Both ARG abundance and the diversity of AMR classes increased. Results provide clear evidence of an increase of ARGs in this remote wild chimpanzee population during a period when ARGs increased regionally and across the globe.

## Introduction

Antimicrobial resistance (AMR) represents a major public health challenge. Although antimicrobial compounds and resistance against them naturally occur in various organisms [1], a concerning increase in clinically relevant AMR is driven by the widespread use of antibiotics [2]. Antimicrobial resistance genes (ARGs) conferring AMR are frequently spread on mobile genetic elements, transferring resistance to new bacteria, creating risks for humans and animals [3]. Close human-animal interactions may facilitate the spread of ARGs among humans, livestock, and potentially wildlife [4]. Bacteria found in wildlife living in close proximity to humans and livestock frequently contain ARGs [5, 6]. Even though ARGs have also been detected in wildlife from more pristine areas, which has been speculated to represent transmission from human contexts with high antibiotic use eg, [7, 8], their origin is often unclear [6]. Longitudinal approaches represent a powerful tool to differentiate between naturally occurring and anthropogenically driven resistance; for example, a 180-year time series of Swedish brown bear oral microbial biofilms showed increases in ARGs coincident with local patterns of human antibiotic use [9]. This study both demonstrates naturally occurring ARGs in bears during the pre-antibiotic era, and an increase in ARGs that coincides with increasing anthropogenic antibiotic use [9].

**Figure 1:**
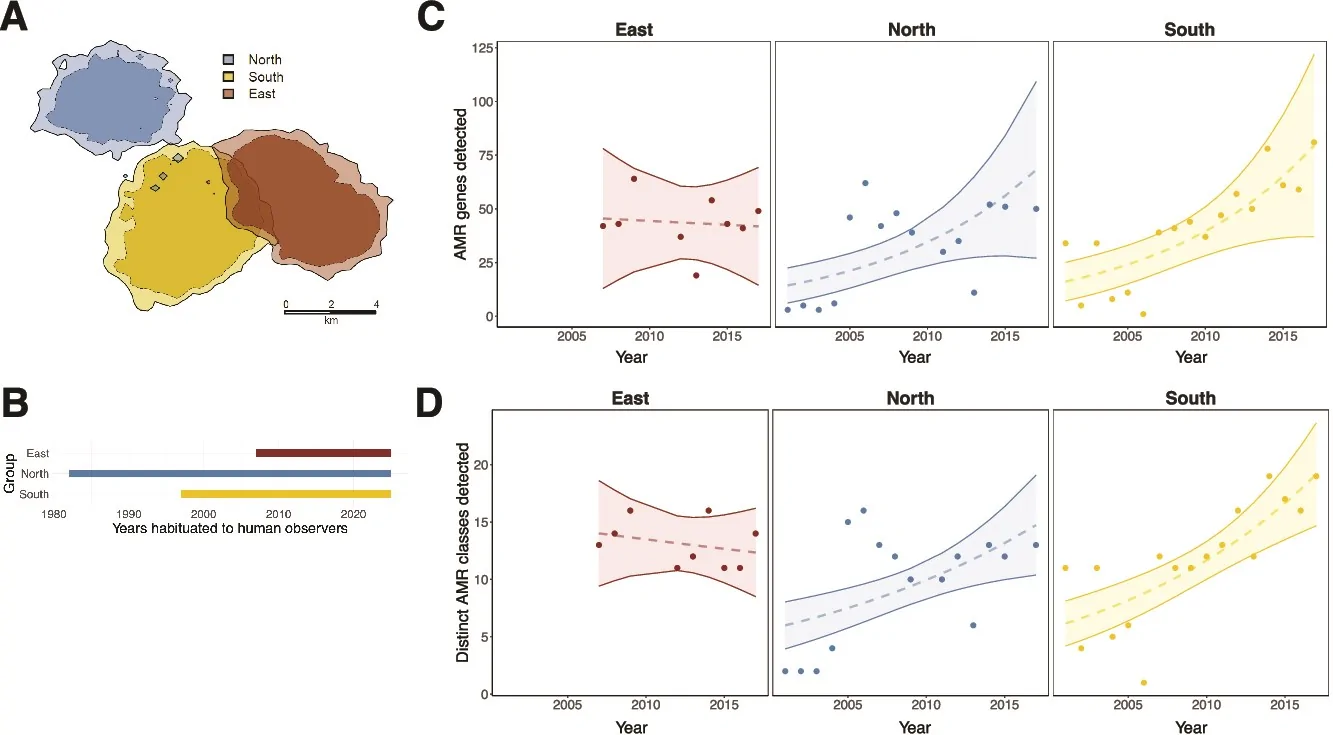
A) the home ranges of the north (blue), south (gold) and east (brown) groups. The black circles indicate the 95% minimum convex polygon. B) the time since the groups have been habituated to human observers. C) the number of ARGs detected in each group through time. D) the number of distinct AMR classes detected in each group in each year. For C, the dashed lines depict the fitted model, and solid lines show the respective 95% confidence limits, for each of the groups. Colors of the raw data points and confidence intervals are as in A.

Here, we employed a longitudinal approach to explore how ARG abundance and diversity changed in wild chimpanzees (*Pan troglodytes verus*) in Taï National Park (TNP), a primary rain forest in Côte d’Ivoire (Fig. 1A). Chimpanzees, one of our closest living relatives, harbor many bacteria similar to those found in humans [10], which likely makes them prone to acquiring ARGs from humans. Though few studies have explored AMR and ARGs in wild chimpanzees and other great apes, some suggest links between increased human contact and higher prevalence of AMR reviewed in: [8].

In TNP, three distinct chimpanzee social groups, the North, South, and East group, have been habituated to human observers by the Taï Chimpanzee Project (TCP) since 1979, 1993, and 2000, respectively [11] (Fig. 1B). Chimpanzees are highly territorial, though there is some minor overlap in the territories of the groups and antagonistic encounters typically occur when groups meet [12]; Fig. 1A. Group size varied throughout the study period (range of non-infant individuals; North = 11 to 18; South = 18 to 42; East = 22 to 34) [13]. Since 2000, fecal samples have been collected regularly and preserved in liquid nitrogen, with long-term storage at −80°C. This created an ideal sample set for molecularly characterizing changes in chimpanzee ARGs [14].

To assess ARG richness and the diversity of resistance types they can confer, we randomly selected a fecal sample from 10 individuals for each group and year, and extracted their DNA. To reduce costs, we pooled equal DNA masses from those samples, and built 41 sequencing libraries (*N*_East_ = 9; *N*_North_ = 15; *N*_South_ = 17 years, representing 410 samples). To assess the potential impact of the long-term sample storage at −80°C on DNA integrity and ensure comparability of sample quality throughout the study period, we performed a TapeStation analysis on a subset of these DNA extracts from the beginning (*N*_extracts_ = 20), middle (*N*_extracts_ = 30), and end of the study period (*N*_extracts_ = 30; Supplementary Material). We found no significant changes in DNA integrity over time, pointing to a broad comparability of sample quality (Study period: *F*_2,69_ = 2.3, *P* = 0.10; group: *F*_2,69_ = 0.6, *P* = 0.57; interaction between group and study period: F_3,69_ = 0.13, *P* = 0.95; Fig. S1 and S2). We only measured the DNA integrity of a subset of the DNA extracts used to build libraries, so we could not include this as a factor in subsequent models.

We used hybridization capture, targeting ARGs from clinically relevant bacteria listed in the Comprehensive Antibiotic Resistance Database [15], and sequenced the captured products. AMR++v3 was used to identify ARGs and assign their class of functional resistances (See: Supplementary Methods). We applied a conservative filter requiring 80% gene coverage to consider a gene present. Many of the ARGs detected are associated with efflux pumps that expel multiple antibiotics, but also play a role in bacterial stress responses, virulence, biofilm formation, and altering bacterial physiology [16]. Consequently, variation in efflux-associated ARG abundance may be influenced by antibiotic exposure, co-selection by other compounds such as metals or biocides, intrinsic bacterial traits, or shifts in community composition [17]. We therefore repeated all analyses using both the full ARG dataset and a reduced dataset excluding efflux-associated ARGs. This reduced analysis was used as a sensitivity analysis to determine whether the observed patterns were driven primarily by broad-specificity efflux-associated genes, or whether similar trends were also present among non-efflux ARGs that are more narrowly associated with antibiotic resistance mechanisms. We found nearly identical results and present results derived from the full dataset (see **Supplementary Results**).

We detected genes associated with resistance to clinically important antibiotics in all three groups (Fig. S5; Table S1). This includes AmpC β-lactamases (*DHA-12, CMY-116/49/63*), responsible for therapeutic failures with third- and fourth-generation cephalosporins and carbapenems; these were first detected in 2006 in the North group, and from then on appeared in 29 pools. Carbapenemase genes (*OXA-211/228/229/230/418*) were first detected in 2008 in the East group and from then on appeared in 26 pools. We sporadically detected *vanA/D/HA/RD/RF* clusters that have been related to vancomycin resistance in nosocomial *Enterococcus* (first detected in 2006 in the North group, and from then on in eight pools), plasmid-mediated quinolone resistance determinants (*QnrB13/48/54/57/73*), and *sul1/2* that can confer fluoroquinolone and sulfamide resistance in *Salmonella* and *E.coli* (first detected in 2006 in the North group, and in 2009 in the East group, respectively, and from then on in each four pools).

The diversity of AMR classes varied across time periods (PERMANOVA full dataset, partial *R*^2^ = 0.29, *P* < 0.001), but not between groups (partial *R*^2^ = 0.036, *P* = 0.508, Fig. S3). We used Generalized Linear models with a poisson or negative binomial error structure (depending on dispersion) and a log link function to test for changes in the number of unique ARGs or the diversity of AMR classes in these groups. For the number of ARGs, there was no interaction between group and time (negative binomial, full dataset; χ^2^ = 3.12, df = 2, *P* = 0.22), but there was a significant increase through time (*P <* 0.001) and no difference between groups (*P =* 0.799, Fig. 1C). For the richness of AMR classes, there was a significant interaction between groups and time (poisson model, full dataset; χ^2^ = 7.27, df = 2, *P* < 0.05; Fig. 1D), with an increase in the diversity of classes and the number of ARGs in both the North and South Groups, but not the East Group, which may reflect the reduced sampling horizon for this group. Both the increases in the number of unique ARGs (negative binomial, full dataset; *P* < 0.001; Fig. S4a) and the diversity of AMR classes were also observed in a model that did not include group as a factor (poisson model, full dataset; *P* < 0.001; Fig. S4b).

Veterinarians at TCP rarely carry out medical interventions. Only in a few cases animals were treated with a long-acting penicillin, which would not create selection pressures that explain observed increases in the number or diversity of ARGs. The increase in ARGs thus points to transmission from the global resistome or exposure to antibiotics themselves. Currently, the route of percolation of ARGs or antibiotics into this ecosystem remains speculative. Although a link to habituation cannot be excluded, other factors such as migratory birds, rain, illegal hunters, or transmission from neighboring unhabituated chimpanzee groups with more contact to humans could play a role [18].

The trade and use of antibiotics in Côte d’Ivoire is largely unregulated, and human mortality attributed to AMR is increasing [4]. The increase in ARG abundance and diversity described here is in line with global and regional trends in the human population, but we do not have data on changes in antibiotics use and ARGs in the local human population and their livestock around TNP, during this period. We were also unable to assess the actual resistance phenotype in the chimpanzee microbiome, and understanding whether the accumulating ARGs translate to increasing phenotypic resistance is an important area of future study [8]. If the accumulation of ARGs in this pristine rainforest does translate into increasing resistance in the bacterial community, this could pose challenges to interventions targeting wildlife. In addition, chimpanzees could serve as mixing vessels for potentially zoonotic bacteria and ARGs given their susceptibility to many of the same pathogens as humans [19–21], and therefore, facilitate the development of resistant zoonotic pathogens.

## Supplementary Material

Tanga_et_al_ISME_Submission_revised_Supplementary_Material2_wrag179

## Data and script availability

Raw sequencing data is available via the European Nucleotide Archive (Project Accession number: PRJEB106947). The reduced CARD database used for the analysis as well as the script for detecting ARGs in the dataset using AMR++ v3.0 v3 are available via Zenodo (10.5281/zenodo.20286184).
