# Supplementary material for: Accumulation of antimicrobial resistance genes in wild chimpanzees
Source: ISME J. 2026 Jul 9;20(1):wrag179. doi: 10.1093/ismejo/wrag179 (PMC13615530; doi:10.1093/ismejo/wrag179)
Supplement: Supplementary file 1 [file tanga_et_al_isme_submission_revised_supplementary_material2_wrag179.docx]

**Supplementary material**

**Accumulation of antimicrobial resistance genes in wild chimpanzees**

Coch Tanguy Floyde Tanga^1,2,3^, Markus Ulrich^3^, J. Benjamin Stielow^3,4^, Elias Eger^3^, Katharina Schaufler^3,5^, Stefan E. Heiden^3^, Michael Schwabe^3^, Liran Samuni^6,7^, Cathy Crockford^7,8^, Tobias Deschner^9,10^, Patrice Makouloutou-Nzassi^1^, Rodrigue Mintsa-Nguema^1^, Roman M. Wittig^7,8^, Sébastien Calvignac-Spencer^2,3^, Fabian H. Leendertz^2,3,7^, Jan F. Gogarten^3,11^*, Ariane Düx^3,7^*

^1^Department of Biology and Animal Ecology, Research Institute for Tropical Ecology (IRET/CENAREST), Libreville BP 13354, Gabon

^2^Faculty of Mathematics and Natural Sciences, University of Greifswald, 17489 Greifswald, Germany

^3^Helmholtz Institute for One Health (HIOH), Helmholtz Centre for Infection Research (HZI), 17489 Greifswald, Germany

^4^Department of Hygiene and Medical Microbiology, Medical University of Innsbruck, 6020-A Innsbruck, Austria

^5^University Medicine Greifswald, 17489 Greifswald, Germany

^6^Cooperative Evolution Laboratory, German Primate Center, 37083 Göttingen, Germany

^7^Taï Chimpanzee Project, Centre Suisse de Recherches Scientifiques, Abidjan, Côte d’Ivoire

^8^The Ape Social Mind Laboratory, Institut des Sciences Cognitives, CNRS, 69500 Bron, France

^9^Comparative BioCognition, Institute of Cognitive Science, University of Osnabrück, 49076 Osnabrück, Germany

^10^Max Planck Institute for Evolutionary Anthropology, 04103 Leipzig, Germany

^11^Department of Applied Zoology and Nature Conservation, Zoological Institute and Museum, University of Greifswald, 17489 Greifswald, Germany

^*^These authors contributed equally.

**Correspondence**: Jan F. Gogarten ([jan.gogarten@helmholtz-hioh.de](mailto:jan.gogarten@helmholtz-hioh.de)) and Ariane Düx ([ariane.duex@helmholtz-hioh.de](mailto:ariane.duex@helmholtz-hioh.de)), Helmholtz Institute for One Health (HIOH), Helmholtz Centre for Infection Research, Fleischmannstrasse 42, Greifswald, Mecklenburg-Vorpommern, 17489, Germany.

**Figure S1:** Gel image showing DNA quality and Integrity Number (DIN) of faecal DNA from five chimpanzees in the EAST, NORTH, and SOUTH groups, collected at multiple time points (EAST: 2007, 2008, 2016, 2017; NORTH: 2001, 2002, 2007, 2008, 2015, 2017; SOUTH: 2001, 2002, 2007, 2008, 2016, 2017) and assessed with the 4200 TapeStation system.

**Figure S2:** Box and whisker plots of the DNA quality and Integrity Number (DIN) for faecal DNA collected at multiple time points during the study period.

**Figure S3.** An ordination (nonmetric multidimensional scaling) of the number of genes detected in different AMR classes for **A**) the full dataset (Sørensen’s dissimilarity, stress = 0.142), and **B**) for the dataset excluding efflux pumps (Sørensen’s dissimilarity, stress = 0.140). Points partially cluster by the time period in which they were collected, but not the group from which they were collected. The color indicates the time period. The shape of the points indicates the chimpanzee group.

**Figure S4.** A) The number of ARGs detected in each group through time, and B) the number of distinct AMR classes detected in each group in each year. The dashed lines depict the fitted model that did not include the social group as a factor, with solid black lines showing the respective 95% confidence limits. Colors of the raw data points as in A.

**Figure S5**. Resistance profiles of the top 15 antibiotic classes and class pairs in the North, South, and East groups based on relative abundance, showing the proportion of antibiotic resistance gene (ARG) detections attributed to each resistance class within each group of pooled samples and for each year (A, C). The number of chimpanzee groups in which the top 15 most abundant genes were detected (B, D). Data shown are from the 80% gene coverage threshold, with panels A and B include efflux pumps, whereas panels C and D exclude efflux pumps. While the MDR resistance shown in this figure is not a class of antibiotics *per se*, it represents the detection of a genes (*abeM, abeS, acrA, acrB, acrD, acrE, acrF, acrS, adeB, adeF, adeG, adeH, adeI, adeJ, adeK, adeS, baeR, bacA, cpxA, cpxR, CRP, emrA, emrB, emrD, emrE, emrK, emrR, emrY, emeA, evgA, evgS, gadX, H-NS, lmrC, lmrD, marA, mdfA, mdtE, mdtF, mdtG, mdtH, mdtK, mdtL, mdtM, mdtN, mdtO, mdtP, mepR, mexB, mexF, mexT, mgrA, nfxB, PmrA, robA, smeB, smeD, smeE, smeF, smeS, tolC*), that are known to confer resistance to more than two classes of antibiotics. The dramatic reduction of MDR detections when excluding efflux pumps, highlights that genes associated with efflux pumps are a major driver of MDR detections seen in A.

**Figure S6.** The number of ARGs detected in each group through time, using the reduced dataset excluding efflux pumps. The dashed lines depict the fitted model, and solid lines show the respective 95% confidence limits, for each of the groups. Colors of the raw data points and confidence intervals are as in Fig. 1.

**Figure S7.** The number of distinct AMR classes detected in each group in each year excluding efflux pumps. The dashed lines depict the fitted model, and solid lines show the respective 95% confidence limits, for each of the groups. Colors of the raw data points and confidence intervals are as in Fig. 1.

**Table S1**. A summary of all genes detected when using an 80% threshold and excluding efflux pumps. Genes associated with multi-drug-resistance (MDR), i.e. resistance to multiple antibiotic families, are highlighted in the legend of Fig. S5.

| **Antibiotic Family** | **Genes** |
| --- | --- |
| Aminoglycosides (enzymatic) | *AAC(6')-Ic, AAC(6')-If, AAC(6')-Iid, AAC(6')-Ij, AAC(6')-Ip, AAC(6')-Ir, APH(3'')-Ib, APH(6)-Id* |
| Aminoglycosides (16S rRNA methylases) | *rmtD, rmtF, rmtG* |
| β-lactams (Ampicillin/Cephalosporins) | *ACT-15, ACT-17, ACT-27, ACT-32, ACT-35, ACT-7, CblA-1, EBR-1, SRT-1, SRT-2 cepA, CFE-1, CMY-116, CMY-49, CMY-63, DHA-12, DHA-18, DHA-20, OXY-2-6* |
| β-lactams (Carbapenemases) | *OXA-211, OXA-212, OXA-228, OXA-229, OXA-230, OXA-235, OXA-257, OXA-355 ,OXA-356, OXA-360, OXA-363, OXA-418* |
| Phenicol/Chloramphenicol | *cat, catB7, catII, catS, cmlA1* |
| Glycopeptides/Vancomycin | *vanA, vanD, vanHA, vanRD, vanRF, vanSF, vanXD, vanYF, vanZA* |
| Macrolides/Lincosamides/Streptogramins (MLS) | *linB, msrE, lsaA, lsaC, lsaE, lnuB* |
| Quinolones/Fluoroquinolones | *QnrB13, QnrB48, QnrB54, QnrB57, QnrB73* |
| Trimethoprim | *dfrA15, dfrE* |
| Sulfonamides | *sul1, sul2* |
| Tetracyclines | *tetX, tetL, tetQ, tetT, tetY, tet39, tet45* |
| Regulators/Biofilm | *baeR, cpxA, cpxR, PmrA, PmrB, PmrC, PmrF, phoP, phoQ, arnA, bacA, evgA, evgS, marA, mgrA, nfxB, robA, nalD* |

**Supplementary Methods:**

***Study population***

The Taï Chimpanzee Project (TCP) was founded in 1979 by Christophe Boesch to carry out research into the behaviour of chimpanzees in Taï National Park. Human contact with the Taï chimpanzees predates the period when these chimpanzees were habituated to human presence [18]. Prior to habituation, interactions with humans were likely predominantly through humans extracting resources in the forest, particularly logging and poaching, and chimpanzees foraging in people’s fields [22, 23]. After the area was designated a protected area in 1972, agro-forestry activities for cocoa production in the northwestern and southwestern peripheries of the study area and use of the forest by village communities for forestry services persisted for some time (although this stopped prior to the start of fecal sampling for this project). Some chimpanzees in the park may have approached these anthropogenically used zones, which includes chimpanzees from the North Group that were observed to raid crops and visit the region during this period.

***Sample collection***

Fecal samples were collected from individually recognizable animals as part of routine health monitoring activities. Each of the three chimpanzee communities are regularly followed by field assistants and researchers who collect behavioral data and fecal samples are opportunistically collected from animals being observed. After watching a known animal defecate, two aliquots of feces are collected as soon as the chimpanzee moves away from the feces, by filling feces into a 2ml Cryotube using the clean end of a stick or a plastic spatula. Upon arrival in camp at the end of the day, tubes are stored in liquid nitrogen until they are shipped to Germany on dry ice and subsequently stored at -80 °C.

***Sample selection and DNA extraction***

Samples from ten different individuals per group per sampling year were randomly selected, resulting in the inclusion of 410 samples in the current study. Nucleic acids were extracted using the Gene MATRIX fecal DNA purification kit (Roboklon, Berlin, Germany) with an extended homogenization procedure involving three freeze-thaw cycles, respectively in liquid nitrogen for 1 min and on a heating block at 70°C for 5 min, followed by cell disruption using FastPrep 4m/s for 20 sec between each cycle. DNA extracts were then stored in -20°C freezers. To assess the integrity of the initial fecal DNA extracts, 20 samples from the EAST group and 30 samples from both the NORTH and the SOUTH were selected spanning the early, middle, and late sampling periods. These were analyzed using the Genomic DNA ScreenTape Assay on the 4200 TapeStation (Agilent Technologies, Santa Clara, CA, USA). Each sample (1 μl DNA + 10 μl buffer) was vortexed (2,000 rpm, 1 min), briefly centrifuged, loaded, and analyzed with the system software.

The DNA extracts were pooled by group and year, using 10 ng of DNA per extract to generate 41 pools that each represented 10 fecal samples and contained 100 ng of DNA. This pooling was carried out to ensure a representative sampling of the microbial community for each sampling year, with gaps in sampling representing periods of civil unrest during which research in the park was drastically reduced. Based on the time of habituation, the groups, and the availability of samples, we created a different total number of pools for the three chimpanzee communities. The East group was represented by nine pools, consisting of extracts from samples collected in 2007, 2008, 2009, 2012, 2013, 2014, 2015, 2016, and 2017. The North group was represented by 15 pools, with samples collected between 2001 and 2017 (2001, 2002, 2003, 2004, 2005, 2006, 2007, 2008, 2009, 2011, 2012, 2013, 2014, 2015, and 2017). For the South group, 17 pools were created, comprising fecal samples collected between 2001 and 2017 (2001, 2002, 2003, 2004, 2005, 2006, 2007, 2008, 2009, 2010, 2011, 2012, 2013, 2014, 2015, 2016, and 2017). This wider temporal range for the South and North group reflects that habituation efforts started earlier for these groups.

***Library preparation***

Pools were concentrated to a volume 50 μl and the DNA was fragmented using the Covaris Ultrasonicator (S220, Shelton, CT, USA), aiming at a fragment length of 350 bp (settings: duration 45 s, peak power 50, duty factor 20%, 1000 cycles per burst, average power 10, temperature 20 °C). During each round of library preparation, a library was also built from chicken DNA, to detect contamination in the laboratory or potential cross contamination between libraries. We prepared libraries with unique dual indices from the 41 pools using the Illumina NEBNext Ultra II kit (New England BioLabs, Ipswich, MA, USA) following the manufacturer's instructions. We performed size selection aiming for 300-400 bp by using AMPure XP magnetic beads (Beckman Coulter, Brea, CA, USA). We performed the indexing PCR for seven cycles under the following conditions: Initial denaturation 98°C for 30s; denaturation 98°C for 10 s; elongation 65°C for 75s; final elongation at 65◦C for 5 min. Indexed libraries were purified with AMPure XP magnetic beads (0.9 ratio) and eluted in 15μl TET. We quantified libraries using the Kapa Library Quantification kit (Roche, Basel, Switzerland) under the following cycling conditions: Initial denaturation 95°C for 5 min; denaturation 95°C for 30 s; elongation 60°C for 45 s; for 35 cycles. Pools below 200ng were selected and purified using the MinElute PCR purification kit (Qiagen, Hilden, Germany), eluted at 15 μl and then a second round of PCR enrichment under the same conditions at 3 cycles was repeated, until all our libraries contained more than 200ng.

***Hybridization capture and high throughput sequencing***

We used a set of 37,826 RNA baits (MYBaits, Daicel Arbor Biosciences, Ann Arbor, MI, USA) targeting over 2,000 genes associated with antibiotic resistance in clinically relevant bacteria [15]. Our 41 chimpanzee and 6 chicken libraries were grouped into four pools, taking 200 ng from each library into the pool. We concentrated these four pools into a final volume of 10µl using the MinElute PCR purification kit. We followed the standard MYBaits Sequence Enrichment kit protocol (Version 5.0), though to reduce costs, only a quarter of the recommended bait quantity was used. Two rounds of hybridization capture were performed for 24 hours at 65°C using biotinylated RNA baits. After the first round of capture, pools were amplified to reach a minimum of 200ng using the KAPA HiFi HotStart ReadyMix (Roche, Basel, Switzerland) and quantified using the KAPA Library Quantification kit. All pools were sequenced on a MiniSeq System (Illumina; high output kit, 2x 150 base paired-end reads).

***Bioinformatic and statistical analysis***

To compare the *DNA quality and Integrity Number* (DIN) of extracts generated from the different groups and years, we used a two-way factorial ANOVA with interaction between the study group and the study period. To detect ARGs in our reads, we used the Comprehensive Antibiotic Resistance Database version 1.0.1 (CARD) that was used to design the capture probes to characterize the ARGs present [24]. CARD provides comprehensive information on resistance genes, mechanisms and antibiotic classes and allows for accurate identification of these genes in complex microbial samples [25]. To reduce sequence redundancy in the CARD database, we employed CD-HIT [26] to cluster similar nucleotide sequences (using a 95% sequence identity threshold), and retained a representative sequence from each cluster. This approach effectively minimized duplication and streamlined the dataset for downstream analyses.

To account for differences in sequencing intensity between libraries, we subsampled our dataset to the minimum number of reads (*N*=146,514 reads) in a given library with seqtk (<https://github.com/lh3/seqtk>) . We then used AMR++ v3.0 v3 [27] to detect ARG in the dataset using the standard_AMR pipeline, removing reads associated with the chimpanzee genome (GCF_028858775.1, NHGRI_mPanTro3-v1.1-hic.freeze_pri) and deduplicated the outcome. We used a gene coverage threshold of 80% to consider that a given antibiotic resistance gene was present.

We used a PERMANOVA implemented in adonis2 in the R package *vegan* [28] to test for the partial impact of social group or years (grouped into three periods; Fig. S1) on AMR class composition, and log transformed the abundance of AMR classes to improve normality.

***Data and script availability***

Raw sequencing data is available via the European Nucleotide Archive (Project Accession number: PRJEB106947). The reduced CARD database used for the analysis as well as the script for detecting ARGs in the dataset using AMR++ v3.0 v3 are available via Zenodo (10.5281/zenodo.20286184)

**Supplementary Results:**

Results consistent with an increase in the number of AMR genes and richness of AMR classes in these groups, were apparent in the dataset excluding efflux pumps as well. The composition of AMR classes varied across time periods (PERMANOVA excluding efflux pumps, partial *R*^2^=0.165, *P*=0.011), but not between groups (partial *R*^2^=0.102, *P*=0.091, **Fig S1**). When excluding the efflux pumps, for the number of unique AMR genes there was as significant interaction between group and time for the full dataset (dispersion of poisson model = 2.55, so we used a negative binomial; χ^2^= 11.39, df=2, *P*<0.005; **Fig. S6A**). For the richness of AMR classes, there was also a significant interaction between the chimpanzee groups and time (dispersion of poisson model = 0.80, so used poisson model; χ^2^= 7.50, df=2, *P*< 0.05; **Fig. S6B**), with an increase in richness and the number of genes in both the North and South Groups, but not the East Group. When excluding the efflux pumps, both the increases in the number of unique ARGs (negative binomial, full dataset; *P*<0.001) and the diversity of AMR classes were also observed in a model that did not include group size as a factor (poisson model, full dataset; *P*<0.001).

**Supplementary references:**

1.	Larsen J, Raisen CL, Ba X *et al*. Emergence of methicillin resistance predates the clinical use of antibiotics. *Nature*. 2022;**602**:135-41 <https://doi.org/10.1038/s41586-021-04265-w>

2.	Murray CJL, Ikuta KS, Sharara F *et al.* Global burden of bacterial antimicrobial resistance in 2019: A systematic analysis. *The Lancet*. 2022;**399**:629-55 <https://doi.org/10.1016/S0140-6736(21)02724-0>

3.	Cazares A, Figueroa W, Cazares D *et al.* Pre- and postantibiotic epoch: The historical spread of antimicrobial resistance. *Science*.**0**:eadr1522 <https://doi.org/doi:10.1126/science.adr1522>

4.	Totaro V, Guido G, Cotugno S *et al.* Antimicrobial resistance in sub-saharan africa: A comprehensive landscape review. *Am J Trop Med Hyg*. 2025;**113**:253-63 <https://doi.org/10.4269/ajtmh.25-0035>

5.	Allen HK, Donato J, Wang HH *et al.* Call of the wild: Antibiotic resistance genes in natural environments. *Nat Rev Microbiol*. 2010;**8**:251-9 <https://doi.org/10.1038/nrmicro2312>

6.	Lee S, Fan P, Liu T *et al.* Transmission of antibiotic resistance at the wildlife-livestock interface. *Communications Biology*. 2022;**5**:585 <https://doi.org/10.1038/s42003-022-03520-8>

7.	Sjölund M, Bonnedahl J, Hernandez J *et al.* Dissemination of multidrug-resistant bacteria into the arctic. *Emerg Infect Dis*. 2008;**14**:70-2 <https://doi.org/10.3201/eid1401.070704>

8.	Tanga CTF, Makouloutou-Nzassi P, Mbehang Nguema PP *et al.* Antimicrobial resistance in African great apes. *Antibiotics*. 2024;**13**:1140

9.	Brealey JC, Leitão HG, Hofstede T *et al.* The oral microbiota of wild bears in Sweden reflects the history of antibiotic use by humans. *Curr Biol*. 2021;**31**:4650-58.e6 <https://doi.org/10.1016/j.cub.2021.08.010>

10.	Rühlemann MC, Bang C, Gogarten JF *et al*. Functional host-specific adaptation of the intestinal microbiome in hominids. *Nature Communications*. 2024;**15**:326 <https://doi.org/10.1038/s41467-023-44636-7>

11.	Boesch C, Wittig R, Crockford C. The chimpanzees of the taï forest: 40 years of research. 2019

12.	Samuni L, Mielke A, Preis A et al. Intergroup competition enhances chimpanzee (*Pan troglodytes verus*) in-group cohesion. *International Journal of Primatology*. 2020;**41**:342-62 <https://doi.org/10.1007/s10764-019-00112-y>

13.	Lemoine S, Boesch C, Preis A *et al*. Group dominance increases territory size and reduces neighbour pressure in wild chimpanzees. *Royal Society Open Science*. 2020;**7**:200577 <https://doi.org/10.1098/rsos.200577>

14.	Gogarten JF, Düx A, Gräßle T *et al.* An ounce of prevention is better : Monitoring wildlife health as a tool for pandemic prevention. *EMBO Rep*. 2024;**25**:2819-31 <https://doi.org/10.1038/s44319-024-00156-z>

15.	Guitor AK, Raphenya AR, Klunk J *et al.* Capturing the resistome: A targeted capture method to reveal antibiotic resistance determinants in metagenomes. *Antimicrob Agents Chemother*. 2019;**64** <https://doi.org/10.1128/aac.01324-19>

16.	Alcalde-Rico M, Hernando-Amado S, Blanco P *et al.* Multidrug efflux pumps at the crossroad between antibiotic resistance and bacterial virulence. *Front Microbiol*. 2016;**7**:1483 <https://doi.org/10.3389/fmicb.2016.01483>

17.	Baker-Austin C, Wright MS, Stepanauskas R et al. Co-selection of antibiotic and metal resistance. *Trends Microbiol*. 2006;**14**:176-82 <https://doi.org/10.1016/j.tim.2006.02.006>

18.	Perry JA, Wright GD. The antibiotic resistance "mobilome": Searching for the link between environment and clinic. *Front Microbiol*. 2013;**4**:138 <https://doi.org/10.3389/fmicb.2013.00138>

19.	Baron SA, Mediannikov O, Abdallah R *et al.* Multidrug-resistant klebsiella pneumoniae clones from wild chimpanzees and termites in Senegal. *Antimicrob Agents Chemother*. 2021;**65**:e0255720 <https://doi.org/10.1128/aac.02557-20>

20.	Calvignac-Spencer S, Leendertz SA, Gillespie TR *et al.* Wild great apes as sentinels and sources of infectious disease. *Clin Microbiol Infect*. 2012;**18**:521-7 <https://doi.org/10.1111/j.1469-0691.2012.03816.x>

21.	Calvignac-Spencer S, Düx A, Gogarten JF *et al.* A great ape perspective on the origins and evolution of human viruses. *Adv Virus Res*. 2021;**110**:1-26 <https://doi.org/10.1016/bs.aivir.2021.06.001>1.	Murray CJL, Ikuta KS, Sharara F *et al.* Global burden of bacterial antimicrobial resistance in 2019: A systematic analysis. *The Lancet*. 2022;**399**:629-55 <https://doi.org/10.1016/S0140-6736(21)02724-0>

22.	Boesch C, Boesch-Achermann H. *The chimpanzees of the taï forest: Behavioural ecology and evolution*: Oxford university press, 2000.

23.	Kablan YA, Diarrassouba A, Mundry R *et al.* Effects of anti-poaching patrols on the distribution of large mammals in taï national park, côte d'ivoire. *Oryx*. 2019;**53**:469-78

24.	Jia B, Raphenya AR, Alcock B *et al.* Card 2017: Expansion and model-centric curation of the comprehensive antibiotic resistance database. *Nucleic acids research*. 2016;**45**:D566-D73 <https://doi.org/10.1093/nar/gkw1004>

25.	Alcock BP, Raphenya AR, Lau TTY *et al.* Card 2020: Antibiotic resistome surveillance with the comprehensive antibiotic resistance database. *Nucleic acids research*. 2019;**48**:D517-D25 <https://doi.org/10.1093/nar/gkz935>

26.	Li W, Godzik A. Cd-hit: A fast program for clustering and comparing large sets of protein or nucleotide sequences. *Bioinformatics*. 2006;**22**:1658-59

27.	Bonin N, Doster E, Worley H *et al.* Megares and amr++, v3.0: An updated comprehensive database of antimicrobial resistance determinants and an improved software pipeline for classification using high-throughput sequencing. *Nucleic acids research*. 2022;**51**:D744-D52 <https://doi.org/10.1093/nar/gkac1047>

28.	Oksanen J, Simpson GL, Blanchet FG *et al.* Vegan: Community ecology package. *(No Title)*. 2001
